# Enhancement of tumor tropism of mPEGylated nanoparticles by anti-mPEG bispecific antibody for ovarian cancer therapy

**DOI:** 10.1038/s41598-021-87271-2

**Published:** 2021-04-07

**Authors:** Wen-Wei Lin, Yi-An Cheng, Chia-Ching Li, Kai-Wen Ho, Huei-Jen Chen, I.-J.u Chen, Bo-Cheng Huang, Hui-Ju Liu, Yun-Chi Lu, Chiu-Min Cheng, Ming-Yii Huang, Hung-Wen Lai, Tian-Lu Cheng

**Affiliations:** 1 Department of Laboratory Medicine, School of Medicine, College of Medicine, Kaohsiung Medical University, Kaohsiung, Taiwan; 2 Graduate Institute of Medicine, College of Medicine, Kaohsiung Medical University, Kaohsiung, Taiwan; 3 Drug Development and Value Creation Research Center, Kaohsiung Medical University, Kaohsiung, Taiwan; 4 Department of Medical Research, Kaohsiung Medical University Hospital, Kaohsiung, Taiwan; 5 Department of Biomedical Science and Environmental Biology, Kaohsiung Medical University, 100 Shih-Chuan 1st Road, Kaohsiung, 80708 Taiwan; 6 Institute of Biomedical Sciences, National Sun Yat-Sen University, Kaohsiung, Taiwan; 7 Department of Aquaculture, National Kaohsiung University of Science and Technology, Kaohsiung, Taiwan; 8 Department of Radiation Oncology, Cancer Center, Kaohsiung Medical University Hospital, Kaohsiung, Taiwan; 9 Endoscopic and Oncoplastic Breast Surgery Center, Comprehensive Breast Cancer Center, Changhua Christian Hospital, 135 Nanxiao Street, Changhua, 500 Taiwan; 10 Division of General Surgery, Changhua Christian Hospital, Changhua, Taiwan; 11 Comprehensive Breast Cancer Center, Changhua Christian Hospital, Changhua, Taiwan; 12 Minimal Invasive Surgery Research Center, Changhua Christian Hospital, Changhua, Taiwan; 13 Department of Laboratory Medicine, Post Baccalaureat Medicine, College of Medicine, Kaohsiung Medical University, Kaohsiung, Taiwan

**Keywords:** Cancer, Cancer imaging, Cancer therapy

## Abstract

Ovarian cancer is highly metastatic, with a high frequency of relapse, and is the most fatal gynecologic malignancy in women worldwide. It is important to elevate the drug susceptibility and cytotoxicity of ovarian cancer cells, thereby eliminating resident cancer cells for more effective therapeutic efficacy. Here, we developed a bispecific antibody (BsAb; mPEG × HER2) that can easily provide HER2^+^ tumor tropism to mPEGylated liposomal doxorubicin (PLD) and further increase the drug accumulation in cancer cells via receptor-mediated endocytosis, and improve the cytotoxicity and therapeutic efficacy of HER2^+^ ovarian tumors. The mPEG × HER2 can simultaneously bind to mPEG molecules on the surface of PLD and HER2 antigen on the surface of ovarian cancer cells. Simply mixing the mPEG × HER2 with PLD was able to confer HER2 specificity of PLD to HER2^+^ ovarian cancer cells and efficiently trigger endocytosis and enhance cytotoxicity by 5.4-fold as compared to non-targeted PLD. mPEG × HER2-modified PLD was able to significantly increase the targeting and accumulation of HER2^+^ ovarian tumor by 220% as compared with non-targeted PLD. It could also significantly improve the anti-tumor activity of PLD (*P* < 0.05) with minimal obvious toxicity in a tumor-bearing mouse model. We believe that the mPEG × HER2 can significantly improve the therapeutic efficacy, potentially reduce the relapse freqency and thereby achieve good prognosis in ovarian cancer patients.

## Introduction

Ovarian cancer ranks as the seventh most common cancer worldwide and the most fatal gynecologic cancer in women with over 22,000 new cases and 14,000 disease-related deaths each year in the United State^1^. These data suggest that almost 55% of all ovarian cancer patients will eventually die from the disease^1^. The high mortality rate of ovarian cancer can be partly ascribed to the majority of patients (approximately 75%) presenting with high-grade disease^2,3^ usually accompanied with metastatic disease in the peritoneal cavity^2^. Although patients can receipt intraperitoneal (IP) chemotherapy (e.g. injected paclitaxel, cisplatin or carboplatin through a catheter) after tumor were optimally debulked and it seems to help some patient live longer than intravenous (IV) chemotherapy alone, however, patients may suffer more severe side effect such as abdominal pain, nausea or vomiting during treatment^1^. Development of a more effective therapeutic strategy for enhancing the therapeutic efficacy of primary or even metastatic ovarian cancer is urgently needed. Methoxyl-polyethylene glycol (mPEG)-labeled liposomal doxorubicin (mPEGylated LD; PLD) is a liposomal nanomedicine that is approved by US Food and Drug Administration (FDA) for clinical use^4–7^. It is most frequently (90.7% of cases) used as a single chemotherapeutic agent or combinatory therapeutic option for the non-platinum treatment of ovarian cancer patients^8–11^. However, lack of targeted and endocytic ability of PLD to cancer cells and the passive diffused cytotoxic mechanism of PLD may make it hard to eliminate all resident cancer cells after primary debulking surgery, thereby elevating the relapse frequency of ovarian cancer^12^ or even inducing severe adverse events that decrease quality of life of ovarian cancer patients during treatment^13^. For example, Gordon et al. suggest that PLD treatment not only failed to prolong the overall survival (OS) rate of platinum-resistant ovarian cancer patients as compared with topotecan treatment but also induced serious adverse events including palmar–plantar erythrodysesthesia (PPE), stomatitis, and mucositis^13–15^. It is important to improve the targeting ability and endocytic efficiency of PLD to target cells for more effective therapeutic efficacy of ovarian cancer.

In this study, we developed a humanized bispecific antibody (BsAb; mPEG × HER2) to easily confer ovarian tumor tropism to PLD. The BsAb consists of one arm of Fab fragment against mPEG, which is lipid anchored on the surface of PLD^5,16–18^, and the other arm of single chain Fv (scFv) fragment against human epidermal growth factor receptor 2 (HER2) antigen, which is overexpressed on approximately 50% of ovarian cancer cells^19–21^. Simply mixing mPEG × HER2 with PLD can easily provide tumor specificity and lead PLD to actively target HER2-overexpressed ovarian tumors and further trigger receptor-mediated endocytosis, thereby enhancing cytotoxicity and therapeutic efficacy in ovarian cancer (Supplementary Figure S1). We first analyzed the functions of both arms of mPEG × HER2 and the HER2 specificity of mPEG × HER2-modified PLD by antigen-based ELISA or cell-based ELISA, respectively. The endocytic ability and efficiency of mPEG × HER2-modified PLD was examined by flow cytometry and confocal live cell imaging. The cytotoxicity of HER2-targeted PLD was further measured by cell viability assay. The tumor accumulation and therapeutic efficacy of mPEG × HER2-modified PLD were finally evaluated in the HER2^+^ ovarian tumor-bearing mouse model. We believe that the increased cytotoxicity of targeted PLD can reduce the opportunity of residual ovarian cancer cell survival from conventional PLD treatment and improve the prognosis of ovarian cancer patients.

## Materials and methods

### Cells and animals

SKOV-3 human ovary adenocarcinoma cells (American Type Culture Collection, Manassas, VA, USA) were cultured in Dulbecco’s modified Eagle’s medium (DMEM; Sigma-Aldrich, St Louis, MO, USA) containing 10% (v/v) fetal bovine serum (FBS; Biological industries, Cromwell, CT, USA) and 100 units mL^−1^ penicillin and streptomycin (Invitrogen, Calsbad, CA, USA), at 37 °C in a humidified atmosphere of 5% (v/v) CO_2_. MDA-MB-468 mammary adenocarcinoma cells (American Type Culture Collection, Manassas, VA, USA) were cultured in DMEM containing 10% (v/v) bovine calf serum (BCS; Thermo, Waltham, MA, USA) and 100 units mL^−1^ penicillin and streptomycin, at 37 °C in a humidified atmosphere of 5% (v/v) CO_2_^22^. Expi293F cells (Thermo, Waltham, MA, U.S.A.) were cultured in Expi293 expression medium (Thermo, Waltham, MA, USA) on shakers (25 mm shaking diameter) with a shake speed of 120 rpm in a humidified atmosphere of 8% CO_2_ in air at 37 °C^22^. Specific pathogen-free 6- to 8-week-old female BALB/c nude mice (BALB/cAnN.Cg-Foxn1nu/CrlNarl) were purchased from the National Laboratory Animal Center, Taipei, Taiwan. All animal procedures were performed in accordance with the Guidelines for Care and Use of Laboratory Animals and approved by the Institutional Animal Care and Use Committee (IACUC) of Kaohsiung Medical University.

### Antibodies and reagents

Hybridomas secreting 6.3 (IgG1 mAbs against PEG) have been described^17^. Monoclonal antibodies were purified from the ascites fluid of mice by affinity chromatography on Protein G Sepharose (GE Healthcare, Little Chalfont, UK) in high-salt buffer. Horseradish peroxidase (HRP)-conjugated goat anti-mouse IgG Fc antibody, HRP-conjugated goat anti-human Fab antibody, HRP-conjugated goat anti-rabbit IgG (H + L) antibody and fluorescein isothiocyanate (FITC)-conjugated goat anti-mouse IgG antibody were from Jackson ImmunoResearch Laboratories (Westgrove, PA, USA). Methoxyl PEGylated-liposomal doxorubicin (PLD; Lipo-Dox) was from Taiwan Tung Yang Biopharm (TTY Biopharm Company, Taipei, Taiwan). Lipo-DiR and Lipo-DiD (PEGylated DOPC/CHOL liposomes labeled with DiR) were purchased from FormuMax Scientific (Sunnyvale, CA, USA).

### Plasmid construction, expression and purification

The construction of mPEG × HER2 and mPEG × DNS was as described in a previous study^23^. In brief, mPEG × HER2 was generated by fusion of the C terminus of an anti-mPEG Fab (clone h15-2b)^17^ with an anti-HER2^23^ or anti-DNS scFv^24^ through a flexible peptide linker (GGGGS)_3_ and mPEG × HER2 and mPEG × DNS were formed, respectively. The V_L_–C_κ_ and V_H_–C_H1_-linker-scFv fragments of mPEG × HER2 or mPEG × DNS were separated with an IRES in the pLNCX retroviral vector (BD Biosciences, San Diego, CA, USA) to generate pLNCX-mPEG × HER2 and pLNCX-mPEG × DNS plasmids, respectively. Expi293 cells were transfected with plasmids and the culture medium was collected after 5 days. The BsAbs were purified by affinity chromatography on methoxyl-PEG_750_ (mPEG_750_)-coated CNBr-activated sepharose 4B (GE Healthcare, Little Chalfont, UK)^23^.

### Enzyme-linked immunosorbent assay (ELISA)

The binding function of anti-mPEG Fab and anti-HER2 scFv arm in mPEG × HER2 was separately analyzed by mPEGylated protein-based ELISA or HER2^+^ cell-based ELISA, respectively. To evaluate the binding ability of anti-mPEG Fab arm, maxisorp 96-well microplates (Nalge Nunc International, Roskilde, Denmark) were coated with 20 μg mL^−1^ of mPEG_2K_-labeled bovine serum albumin (BSA) in 50 μL well^−1^ of 0.1 M NaHCO_3_ (pH 9.0) at 37 °C for 2 h and then blocked with 200 μL well^−1^ of dilution buffer [5% (wt/vol) skim milk in PBS] overnight at 4 °C. Serial dilutions of mPEG × HER2 or mPEG × DNS were added to the wells at room temperature (RT) for 45 min. After extensive washing, the bound BsAb was detected by HRP-conjugated anti-human Fab secondary Ab. The plates were washed with PBS, and bound peroxidase activity was measured by adding 150 μL well^−1^ of ABTS solution [0.4 mg mL^−1^, 2′-azinobis (3-ethylbenzthiazoline-6-sulfonic acid), 0.003% (v/v) H_2_O_2_, and 100 mM phosphate-citrate, pH 4.0] for 40 min at RT. Color development was measured at 405 nm on a EZ Read 400 ELISA (Biochrom). The binding ability of the anti-HER2 scFv arm in mPEG × HER2 was examined by HER2^+^ cell-based ELISA. HER2^+^ SKOV-3 cells (2 × 10^5^) were seeded in poly-d-lysine (50 μg mL^−1^, Corning, New York, USA)-coated 96-well cell culture plates overnight at 37 °C. After washing, the cells were fixed with 2% (w/v) paraformaldehyde for 5 min at RT and the reaction was stopped by the neutralization of 0.1 M glycine solution at RT for 2 h. Serial dilutions of mPEG × HER2 or mPEG × DNS were added to each well and reacted at RT for 45 min. After extensive washing with PBS, 10 μg mL^−1^ of mPEG_2K_-BSA was added to the wells for 20 min. After washing, the bound mPEG_2K_-BSA was detected by subsequent addition of 5 μg mL^−1^ 6.3 anti-PEG backbone Ab for 1 h, and 0.4 μg mL^−1^ HRP-conjugated goat anti-mouse IgG Fc. The plates were washed with PBS, and bound peroxidase activity was measured by adding 150 μL well^−1^ of ABTS solution (pH 4.0) and 0.003% (v/v) H_2_O_2_ for 40 min at RT. Color development was measured at 405 nm on a EZ Read 400 ELISA. In order to examine whether the mPEG × HER2 can simultaneously bind to mPEG and HER2 antigen, mPEG × HER2- or mPEG × DNS-modified PLD were incubated with SKOV-3 (HER2^+^) or MDA-MB-468 (HER2^-^)-seeded 96-well culture plate, followed by staining with 5 μg mL^−1^ 6.3 anti-PEG backbone Ab for 1 h, and 0.4 μg mL^−1^ HRP-conjugated goat anti-mouse IgG Fc. The absorption value of HRP reaction was detected by the same procedure as mentioned above.

### Flow cytometry

Internalization of mPEG × HER2-modified nanoparticles into HER2^+^ cancer cells was analyzed by adding 2 μg mL^−1^ of Lipo-DiD, mPEG × DNS-modified Lipo-DiD or mPEG × HER2-modified Lipo-DiD in staining buffer (PBS containing 0.05% (w/v) BSA) to 2 × 10^5^ SKOV-3 cells for 40 min at 4 °C. After extensive washing, the cells were transferred to fresh culture medium and incubated for 1, 6 and 12 h at 37 °C. The surface BsAb-modified Lipo-DiD on the SKOV-3 cells was determined by sequentially adding 10 μg mL^−1^ 6.3 anti-PEG Ab for 30 min and 4 μg mL^−1^ FITC-conjugated goat anti-mouse IgG Fcγ. After washing, the FITC signal was measured with a Cytomics FC500 flow cytometer (Beckman Coulter, CA, USA).

### Western blot

SKOV-3 cancer cells (2 × 10^5^) were seeded in a 96-well cell culture plate at 37 °C overnight. The next day, the cells were incubated with serum free medium (Control), 8 μg mL^−1^ of PLD, mPEG × HER2 BsAb, mPEG × HER2-, mPEG × DNS-modified PLD or 80 ng mL^−1^ doxorubicin at 37 °C for 24 h. The cells were extracted with radioimmune precipitation assay (RIPA) buffer and an equal amount of proteins were separated on SDS-PAGE and then transferred to NC membranes. The membrane was blocked with PBS containing 5% (w/v) skim milk (BD) and incubated with specific primary antibodies against poly (ADP-ribose) polymerase (PARP), cleaved caspase 9 or β-actin, respectively, for 1 h. After washing, appropriated HRP-conjugated goat anti-mouse IgG Fc (Jackson ImmunoResearch Laboratories, Westgrove, PA, USA) or HRP-conjugated goat anti-rabbit IgG (H + L) (Jackson ImmunoResearch Laboratories, Westgrove, PA, USA) were stained and incubated for 1 h at room temperature (RT), and expression of protein was detected using an ECL kit (Millipore, Temecula, CA, USA) and ChemiDoc MP Imaging System (Bio-red). The density of each band were quantified by ImageJ software.

### Confocal microscopy of mPEG × HER2-modified PLD

To analyze the internalization of mPEG × HER2-modified PLD into HER2 + cancer cells, we seeded 2 × 10^5^ SKOV-3 cells on 2 μg mL^−1^ poly-l-lysine-coated glass slides at 37 °C in a humidified atmosphere of 5% CO_2_ for 24 h. The cells were incubated with 0.5 mg mL^−1^ Hoechst 33,342 (Invitrogen, Carlsbad, CA, USA) and 50 nmol of LysoTracker Green DND-26 in DMEM to stain the nucleus and lysosomes, respectively, for 40 min at 37 °C. After extensive washing with fresh culture medium, the cells were stained with 0.01 nmol of mPEG × DNS-modified Lipo-DiD or mPEG × HER2-modified Lipo-DiD in fresh culture medium at 37 °C. The fluorescence signals were monitored in real time with a Zeiss LSM780 laser-scanning microscope (Carl Zeiss AG, Oberkochen, Germany). To detect the PLD accumulated in tumor tissue, the SKOV-3 tumor-bearing BALB/c nude mice were separately and intravenously injected with 5 mg kg^−1^ of PLD, mPEG × DNS-modified PLD or mPEG × HER2-modified PLD. Tumors were collected at 48 h post-injection and embedded in Tissue-Tek OCT Compound (Sakura Finetek USA, Torrance, CA, USA) at − 80 °C overnight. The nuclei were stained with DAPI fluoromount-G (Southern Biotech, Birmingham, AL, USA) and the fluorescent signals of doxorubicin and DAPI were observed under an Olympus FluoView FV1000 confocal microscope (Olympus Imaging America, Shinjuku City, Tokyo, Japan).

### In vitro cytotoxicity

SKOV-3 cancer cells (3 × 10^3^) were seeded in a 48-well cell culture plate at 37 °C overnight. The next day, the cells were incubated with serum free medium (Control) or serial dilutions of PLD, mPEG × DNS-modified PLD or mPEG × HER2-modified PLD (100 μL well^−1^) at 37 °C for 12 h and then the medium was replaced with fresh medium. The cell viability was measured with the ATPlite luminescence assay system (PerkinElmer, Waltham, MA) 96 h post drug treatment. As previous study described^23^, the results are expressed as percentage inhibition of luminescence as compared with untreated cells and calculated by the following formula: % cell viability = 100 × (treated luminescence/untreated luminescence). The standard deviation for each point was averaged over four samples (n = 3).

### In vivo optical imaging

SKOV-3 tumor-bearing BALB/c nude mice were separately and intravenously injected with 10 nmol mPEG × DNS-modified Lipo-DiR or mPEG × HER2-modified Lipo-DiR when the tumor size was 200 mm^3^. The fluorescent signal of Lipo-DiR was monitored by IVIS spectrum optical imaging system (excitation, 750 nm; emission, 780 nm; PerkinElmer, Waltham, MA, USA) at 24, 48 and 72 h post-injection. The tumors and different organs (liver, spleen, heart, lung, ovary and kidney) of each group were collected at 72 h after Lipo-DiR injection. As previous study described^23^, the region of interest (ROI) in tumors or different organ areas were drawn and analyzed with Living Image software version 4.2 (Caliper Life Sciences).

### In vivo antitumor therapy

SKOV-3 tumor-xenografted BALB/c nude mice were intravenously injected with saline, 5 mg kg^−1^ PLD, mPEG × HER2 BsAb, mPEG × HER2- or mPEG × DNS-modified PLD when the tumor size was 150 mm^3^, once a week for 4 weeks. The tumor volume was monitored using calipers every 3 days post-treatment and the tumor sizes were calculated using the following formula: volume = (length × width^2^)/2. The body weight of the mice in each group was measured twice a week post-treatment.

### Statistical analysis

Statistical significance of differences between mean values was estimated by GraphPad Prism v.6 (GraphPad Software, San Diego, CA, U.S.A.) using the repeated measures unpaired *t* test. Data were considered significant at a P value of less than 0.05.

## Results

### Dual function of humanized BsAb (mPEG × HER2)

**Figure 1:**
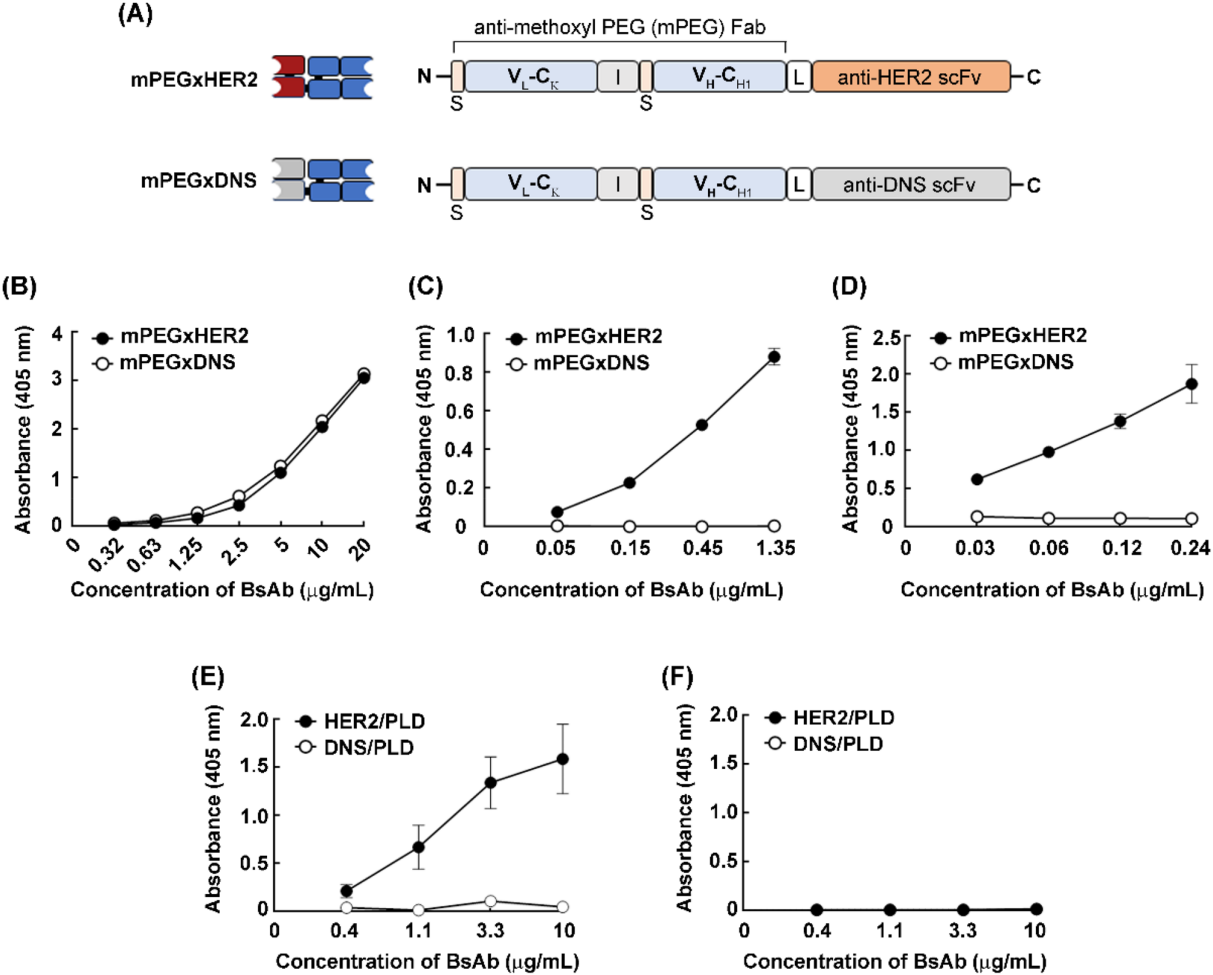
Characterization of mPEG × HER2. (**A**) The gene constructs of mPEG × HER2 and mPEG × DNS are composed of a signal peptide (S), the anti-mPEG V_L_–C_κ_, the anti-mPEG V_H_–C_H1_, a flexible linker peptide (L) and an anti-HER2 scFv (mPEG × HER2) or control anti-DNS scFv (mPEG × DNS). I, internal ribosomal entry site (IRES). The (**B**) mPEG and (**C**) HER2 binding functions of mPEG × HER2 (filled circle) or mPEG × DNS (open circle) on mPEGylated protein and HER2^+^ cancer cells were detected via ELISA (n = 2). Bars, SD. (**D**) The simultaneous binding ability of mPEG and HER2 antigen of mPEG × HER2 (filled circle) and mPEG × DNS (open circle) were analyzed by targeting SKOV-3 (HER2^+^) cells and detected via cell-based ELISA (n = 4). Bars, SD. The specific targeting ability of mPEG × HER2^−^ (filled circle, HER2/PLD) and mPEG × DNS-modified PLD (open circle, DNS/PLD) were analyzed by targeting (**E**) SKOV-3 (HER2^+^) or (**F**) MDA-MB-468 (HER2^−^) cells and detected via cell-based ELISA (n = 4). Bars, SD.

**Figure 2:**
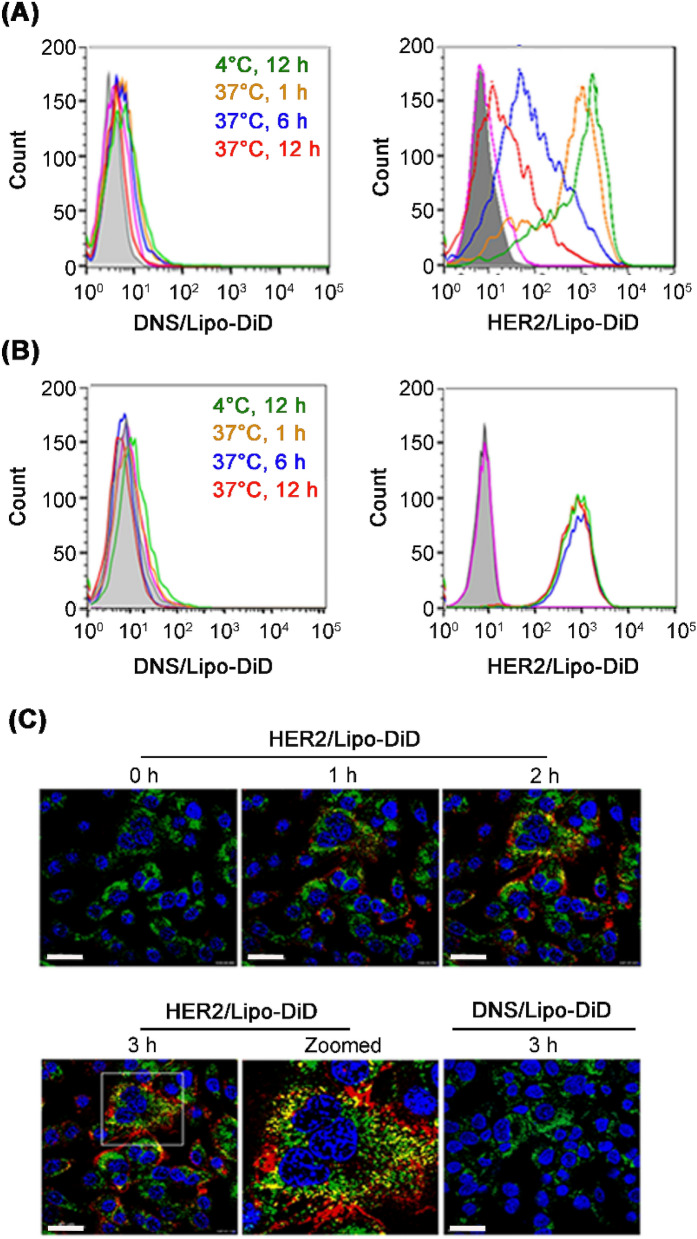
mPEG × HER2 improves the internalization of mPEGylated nanoparticles into HER2^+^ ovarian cancer cells. The internalization ability of mPEG × HER2-modified nanoparticles was performed by incubating mPEG × DNS-modified Lipo-DiD (DNS/Lipo-DiD) or mPEG × HER2-modified Lipo-DiD (HER2/Lipo-DiD) to HER2^+^ SKOV-3 cells at 4 °C for 12 h (green), 37 °C for 1 (orange), 6 (blue) and 12 h (red) and the surface bound mPEGylated nanoparticles were detected by staining with 6.3 anti-PEG antibody and FITC-conjugated secondary Ab and analyzed by flow cytometry. The internalized Lipo-DiD in SKOV-3 cells were evaluated by monitoring the (**A**) decreasing level of surface FITC signal. The (**B**) total mPEGylated nanoparticles (including surface and internalized Lipo-DiD) were monitored by detecting the red fluorescent signal of Lipo-DiD. The gray peak on the graphs show mock staining with PBS containing 0.05% (wt/vol) BSA. (**C**) The internalization process of mPEG × HER2-modified Lipo-DiD was investigated by confocal live cell imaging system. The HER2^+^ SKOV-3 cells were stained with Hoechst 33342 (blue), LysoTracker (green) and mPEG × DNS^−^ (DNS/Lipo-DiD) or mPEG × HER2-modified Lipo-DiD (HER2/Lipo-DiD) (red). The co-localization of Lipo-DiD with lysosome (yellow) in SKOV-3 cells was observed in real time by confocal microscopy. Scale bar 40 μm.

To generate a BsAb that can simultaneously bind to mPEG and HER2 antigen, we constructed mPEG × HER2 as described in our previous study^23^. We fused a Fab fragment of the humanized anti-mPEG antibody 15-2b, which can specifically target the methoxy-end of mPEG^17^ to an anti-HER2 scFv by linking with a flexible linker (Fig. 1A). The mPEG × DNS was also constructed as a negative control by replacing the anti-HER2 scFv to anti-dansyl (DNS) scFv, which can bind to a small chemical hapten dansyl^25^ that is not present on the surface of cancer cells. We further analyzed the antigen binding ability of mPEG × HER2 and mPEG × DNS by mPEG-based or HER2^+^ cell-based ELISA, respectively. Figures 1B and 2C show that mPEG × HER2 and mPEG × DNS can both bind to the mPEG_2K_ molecule but only mPEG × HER2 can specifically bind to HER2 antigen on the surface of HER2^+^ ovarian cancer cells (SKOV-3)(Fig. 1C). To examine whether the mPEG × HER2 can simultaneously binding to mPEG and HER2 antigen, sandwich cell-based ELISA was performed by immobilizing SKOV-3 (HER2^+^) cells on a 96-well cell culture plate, followed by subsequent incubation with mPEG × HER2 or mPEG × DNS, mPEG_2K_-BSA, anti-PEG monoclonal Ab 6.3 and HRP-conjugated secondary Ab. Figure 1D shows that only mPEG × HER2- but not mPEG × DNS can simultaneously bind to SKOV-3 cells and mPEGylated molecules (i.e., mPEG_2K_-BSA) in a dose-dependent manner (Fig. 1D). In order to examine whether the mPEG × HER2 can confer PLD to HER2 specificity, sandwich cell-based ELISA was performed by immobilized SKOV-3 (HER2^+^) or MDA-MB-468 (HER2^−^) cells into a 96-well cell culture plate, followed by subsequent incubation with mPEG × HER2^−^ or mPEG × DNS-modified PLD, anti-PEG monoclonal Ab 6.3 and HRP-conjugated secondary Ab. Figure 1E shows that only mPEG × HER2^−^ but not mPEG × DNS-modified PLD can bind to SKOV-3 cells in a dose-dependent manner (Fig. 1E) and neither mPEG × HER2^−^ nor mPEG × DNS-modified PLD was bound to MDA-MB-468 cells (Fig. 1F). These results indicate that mPEG × HER2 can simultaneously recognize the mPEG molecule and HER2 antigen, provide HER2 tropism to PLD, and specifically target to HER2-overexpressed ovarian cancer cells.

### Internalization of mPEG × HER2-modified PLD into HER2^+^ cancer cells via receptor-mediated endocytosis

To investigate the internalization ability and efficiency of mPEG × HER2-modified PLD to HER2^+^-overexpressed ovarian cancer cells, we modified mPEG × HER2 or mPEG × DNS to Lipo-DiD, which is a mPEGylated nanoparticle that can exert red fluorescent signals after excitation, and tracked the fluorescent signals after incubating with HER2^+^ SKOV-3 cells for different time periods (1, 6 and 12 h) by flow cytometry and live cell imaging system. Figure 2A shows that the mPEG × HER2-modified Lipo-DiD on HER2^+^ the ovarian cancer cell surface was gradually decreased in a time-dependent manner after incubation with Lipo-DiD and no surface Lipo-DiD was detected in the mPEG × DNS-modified Lipo-DiD group (Fig. 2A). In contrast, the total Lipo-DiD signal obtained from different compartments of cells were similar at different time points (Fig. 2B), indicating that the initial targeted level of mPEG × HER2^−^ or mPEG × DNS-modified Lipo-DiD to SKOV-3 cells were almost the same in each corresponding groups. These results indicate that mPEG × HER2-modified Lipo-DiD can specifically target HER2^+^ ovarian cancer cells and efficiently trigger endocytosis within 6 h. In live cell imaging, the mPEG × HER2-modified Lipo-DiD was incubated with HER2^+^ SKOV-3 for different time periods (1–3 h) and the fluorescence of lysosomes (green), Lipo-DiD (red) and co-localized signal (yellow) were observed by real-time confocal microscopy to monitor its binding, internalization and location in cells. Figure 2C shows that the bound mPEG × HER2-modified Lipo-DiD was internalized at 1 h and fused with lysosomes (yellow signal) within 2 h and the co-localized signal (yellow) was gradually increased in a time-dependent manner post-incubation; however, no Lipo-DiD signal was detected in the mPEG × DNS-modified Lipo-DiD group (Fig. 2C). We further analyzed the internalization ability of different dosage of mPEG × HER2-modified Lipo-DiD to HER2^+^ SKOV-3 cells, we tracked the fluorescent signals after incubating different concentration of mPEG × HER2-modified Lipo-DiD (4 or 8 μg mL^−1^) with HER2^+^ SKOV-3 cells for 12 h by flow cytometry. The results showed that the cell surfaced mPEG × HER2-modified Lipo-DiD on SKOV-3 cells were gradient increased in dose-dependent manner and they can all be internalized into the cells after transferred the BsAb-modified Lipo-DiD-labeled SKOV-3 cells from 4 to 37 °C for 12 h (Supplementary Figure S2). These results suggest that mPEG × HER2-modified nanoparticles can trigger efficient internalization and accumulation via receptor-mediated endocytosis in HER2-overexpressed ovarian cancer cells.

### Cytotoxicity of mPEG × HER2-modified PLD to HER2-overexpressed ovarian cancer cells

**Figure 3:**
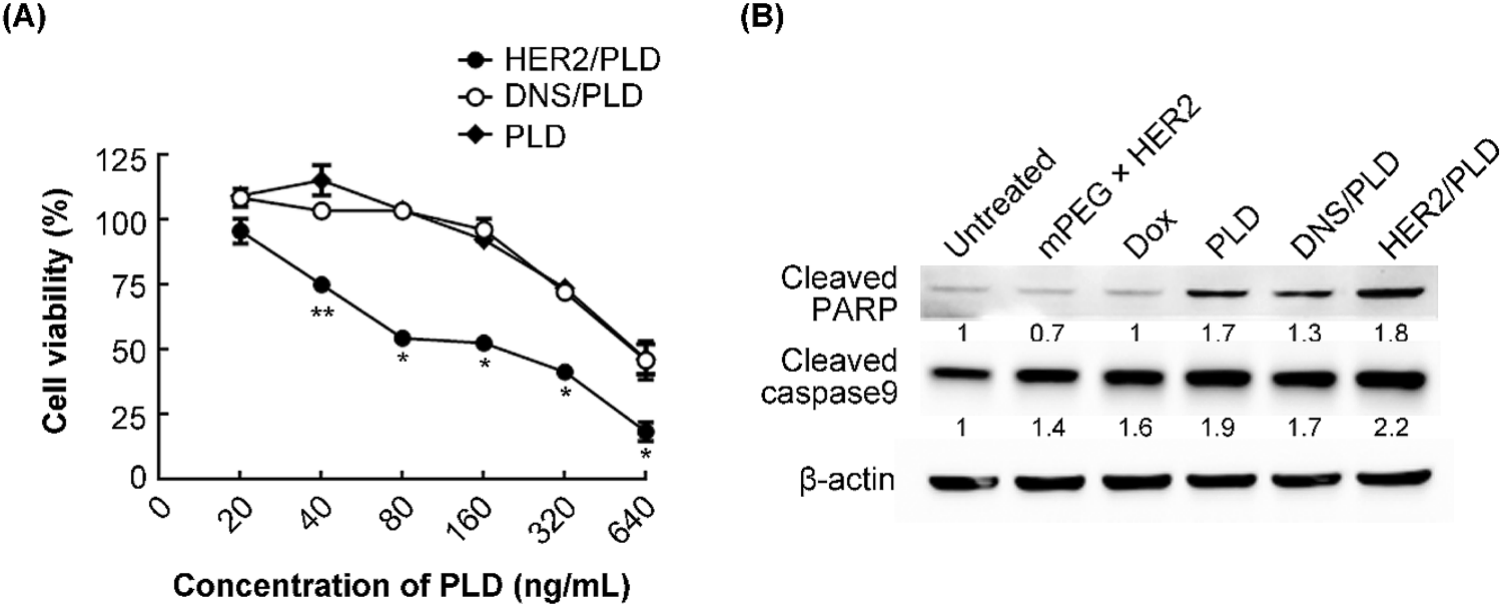
mPEG × HER2 enhances the cytotoxicity of PLD to HER2^+^ ovarian cancer through triggering apoptosis pathway. (**A**) PLD (filled diamond), mPEG × DNS-modified PLD (open circle, DNS/PLD) or mPEG × HER2-modified PLD (filled circle, HER2/PLD) were incubated with SKOV-3 ovarian cancer cells for 12 h. The cell viability was determined by ATPlite analysis and the mean luminescence values compared to untreated control cells (n = 3). Bars, SD. *P < 0.05. **P < 0.01. (**B**) mPEG × HER2, Dox, PLD, DNS/PLD or HER2/PLD were incubated with SKOV-3 ovarian cancer cells for 24 h. The apoptotic marker proteins (i.e. PARP and Caspase 9) were analyzed by Western blot and the relative ratio of apoptotic marker proteins to β-actin is shown below each lane. The cell lysates of each treated group were analyzed as described above. Lane 1, untreated group was used as a negative control. Dox, doxorubicin.

In order to analyze the cytotoxicity of mPEG × HER2-modified PLD to HER2-overexpressed ovarian cancer cells, we incubated HER2^+^ SKOV-3 cells with different concentrations of PLD, mPEG × HER2^−^ or mPEG × DNS-modified PLD for 12 h, followed by removing the drug, and washing the cells. The cell viability was determined by ATPlite assay 96 h post-drug addition. Figure 3A indicates that mPEG × HER2-modified PLD (IC_50_ = 160 ng mL^−1^) significantly reduced the cell viability of SKOV-3 from a drug concentration of 40 ng mL^−1^ as compared with mPEG × DNS-modified PLD (IC_50_ = 870 ng mL^−1^) and the PLD group (IC_50_ = 900 ng mL^−1^) and showed approximately 5.4-fold enhanced toxicity in comparison with non-targeted PLD (Fig. 3A). As expected, there was no difference between mPEG × DNS-modified PLD and unmodified PLD (Fig. 3A). We also compared the cytotoxicity of mPEG × HER2 BsAb and anti-HER2 antibody (i.e. Trastuzumab) to SKOV-3 cell line. Both of mPEG × HER2 BsAb and Trastuzumab were showed only 10% reduced viability of SKOV-3 cell line (Supplementary Figure S3), indicating that the significantly elevated toxicity of HER2/PLD was contributed by the specific targeting ability of mPEG × HER2 BsAb. To study whether the enhanced cytotoxicity of mPEG × HER2-modified PLD is through inducing stronger apoptosis pathway, we treated 8 μg mL^−1^ PLD, mPEG × HER2 BsAb, mPEG × HER2^−^, mPEG × DNS-modified PLD or 80 ng mL^−1^ doxorubicin for 24 h and the apoptotic marker proteins [i.e. poly (ADP-ribose) polymerase (PARP) and caspase 9] were further be detected by western blot. Figure 3B shows that the PARP and caspase 9 were all increased in mPEG × HER2-modified PLD-treated group as compared with mPEG × DNS-modified PLD or PLD group. The expression level of apoptotic marker proteins (PARP and Caspase 9) in mPEG × HER2 BsAb-treated group showed no significant difference as compared with untreated group (Fig. 3B). These results suggest that the mPEG × HER2-modified PLD improved the cytotoxicity of PLD in HER2-overexpressed ovarian cancer cells through triggering stronger apoptosis pathway.

### Specific targeting and accumulation of mPEG × HER2-modified PLD to HER2^+^-overexpressed tumors

**Figure 4:**
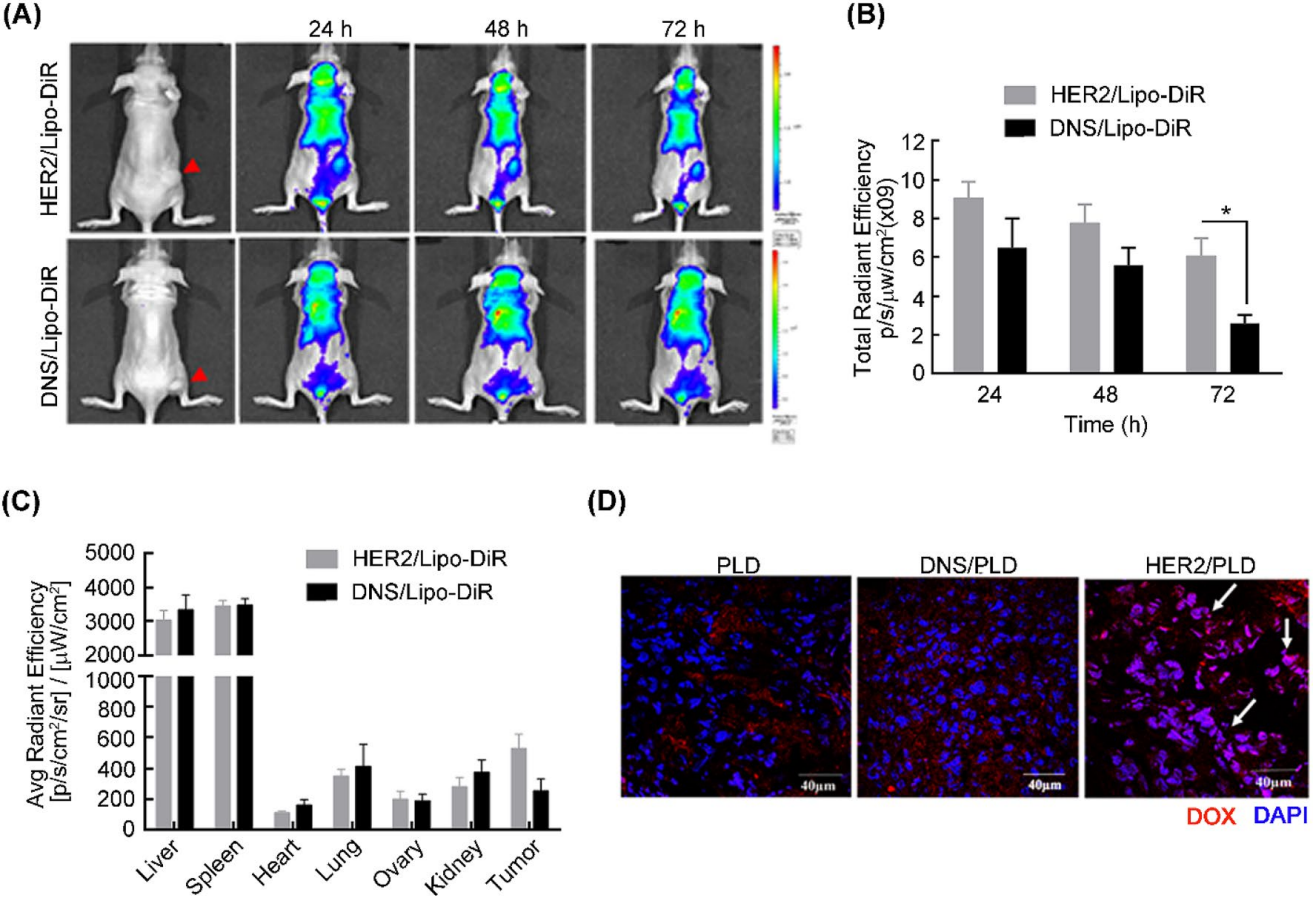
mPEG × HER2 improves the tumor targeting and accumulation of PLD in ovarian tumor tissue. (**A**) mPEG × DNS^−^ (DNS/Lipo-DiR) or mPEG × HER2-modified Lipo-DiR (HER2/Lipo-DiR) were intravenously injected in SKOV-3 tumor-bearing nude mice (red arrow at right flank). The fluorescence intensity of DiR was monitored at 24, 48 and 72 h post-injection by IVIS spectrum optical imaging system. (**B**) The total region of interest (ROI) in SKOV-3 tumors that were treated with mPEG × DNS^−^ (black, DNS/Lipo-DiR) or mPEG × HER2-modified Lipo-DiR (gray, HER2/Lipo-DiR) was quantified by Living Image software. (**C**) The total ROI of collected tumors and different organs (liver, spleen, heart, lung, ovary and kidney) treated with mPEG × DNS^−^ (black, DNS/Lipo-DiR) or mPEG × HER2-modified Lipo-DiR (gray, HER2/Lipo-DiR) were quantified at 72 h (n = 5). Bars, SEM. *P < 0.05. (**D**) The accumulated effect of doxorubicin of HER2-targeted PLD in ovarian tumor was measured by treating PLD, mPEG × DNS-modified PLD (DNS/PLD) or mPEG × HER2-modified PLD (HER2/PLD) to SKOV-3 tumor-bearing nude mice and detecting the fluorescent signal of doxorubicin at 48 h after treatment. The tumors were collected and the nucleus stained by DAPI. The fluorescent signal of doxorubicin (red) and DAPI (blue) were detected by confocal microscopy and the co-localized signals of doxorubicin and nucleus in HER2/PLD-treated mice are indicated (white arrow). Scale bar 40 μm.

To investigate the specific targeted ability and accumulated efficiency of mPEG × HER2-modified nanoparticle drug to HER2-overexpressed ovarian tumor in vivo, we established a human ovarian carcinoma-xenografted model by subcutaneously inoculating HER2^+^ SKOV-3 cells at the hind foot of nude mice and intravenously treating with mPEG × HER2^−^ or mPEG × DNS-modified far red-labeled liposomes (PEGylated liposomal DiR; Lipo-DiR), respectively, when tumor size had grown to 200 mm^3^. The red fluorescent signal of Lipo-DiR was detected by IVIS imaging system at 24, 48 and 72 h post-injection. Figure 4A shows that the mPEG × HER2-modified Lipo-DiR can more specifically target to HER2^+^ ovarian tumors as compared with mPEG × DNS-modified Lipo-DiR. The quantitative signal of the region of interest (ROI) of mPEG × HER2-modified Lipo-DiR-treated tumors was increased by 140% (9.1 × 10^9^ versus 6.5 × 10^9^), 140% (7.8 × 10^9^ versus 5.6 × 10^9^) and 240% (6.1 × 10^9^ versus 2.6 × 10^9^) at 24, 48 and 72 h, respectively, as compared with mPEG × DNS-modified Lipo-DiR (Fig. 4B). The mice were further sacrificed and the organs (liver, spleen, heart, lung, ovary and kidney) were collected at 72 h post-injection to monitor the biodistribution of targeted Lipo-DiR. Figure 4C indicates that the targeted Lipo-DiR was preferentially accumulated in tumor tissue and non-specifically accumulated in liver and spleen tissue, which are major metabolic organs for nanoparticle drugs^26,27^. In order to evaluate whether mPEG × HER2-modified PLD can efficiently enter into the nucleus of HER2^+^ ovarian tumor cells, we further intravenously treated PLD, mPEG × HER2- or mPEG × DNS-modified PLD to SKOV-3-xenografted nude mice and collected the tumor tissue for detecting the doxorubicin signal at 48 h after drug treatment. As shown in Fig. 4D, there was more red fluorescence of doxorubicin signal accumulated at the HER2^+^ ovarian tumor region and colocalized with the nucleus of tumor cells than for the mPEG × DNS-modified PLD or PLD control group. These results indicate that mPEG × HER2-modified nanoparticles can more efficiently target and accumulate to HER2^+^ ovarian tumor than non-targeted PLD.

### Therapeutic efficacy of mPEG × HER2-modified PLD in HER2^+^ tumor-xenografted nude mice

**Figure 5:**
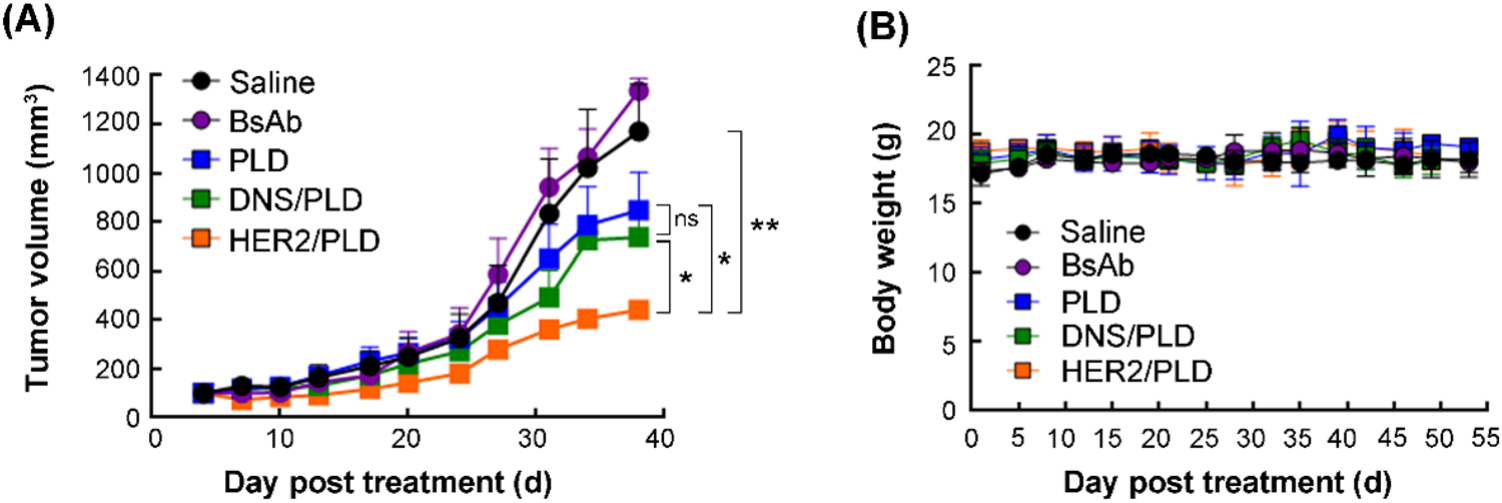
mPEG × HER2 improves the therapeutic efficacy of PLD HER2-overexpressed ovarian cancer. We injected 5 mg kg^−1^ of saline (black circle, n = 3), PLD (blue square, n = 4), mPEG × HER2 BsAb (violet circle, n = 3), mPEG × HER2^−^ (yellow square, HER2/PLD, n = 4) or mPEG × DNS-modified PLD (green square, DNS/PLD, n = 4) into SKOV-3 tumor-bearing nude mice when tumor size was 150 mm^3^, once a week for 4 weeks. (**A**) The tumor volume of each treatment and (**B**) the body weight were measured every 3 days post-treatment. Bars, SEM. **P < 0.01. *P < 0.05. *ns* not significant.

To investigate the therapeutic efficacy of mPEG × HER2-modified PLD to HER2-overexpressed ovarian tumor in vivo, we established SKOV-3 tumor-bearing nude mice and intravenously treated them with saline, 5 mg kg^−1^ PLD, mPEG × HER2 BsAb, mPEG × HER2^−^ or mPEG × DNS-modified PLD once a week for 4 weeks and the tumor volume and body weight of tumor-bearing mice was measured twice a week. Figure 5A shows that the mPEG × HER2-modified PLD can significantly inhibit the HER2^+^ ovarian tumor growth as compared to treatment with mPEG × DNS-modified PLD (*P* = 0.0319) or PLD (*P* = 0.0437) and there was no significant difference between mPEG × DNS-modified PLD- and PLD-treated groups (*P* = 0.5754) (Fig. 5A). There was no significant change in the body weight of each treated group (Fig. 5B). These results demonstrate that mPEG × HER2-modified PLD can enhance the therapeutic efficacy of PLD to HER2-overexpressed ovarian cancer with minimal obvious toxicity.

## Discussion

In this study, we successfully developed a BsAb (mPEG × HER2) which can simultaneously recognize mPEG molecules on the surface of mPEGylated nanomedicine (i.e., PLD) and the HER2 antigen on the surface of ovarian cancer cells, and easily provide HER2^+^ tumor specificity to PLD by a simple mixing procedure. We demonstrated that the mPEG × HER2-modified PLD can significantly enhance its tumor targeted specificity, endocytic efficiency and cytotoxicity to HER2-overexpressed SKOV-3 cells and also increase its accumulation and anti-cancer activity in a HER2^+^ tumor-xenografted mouse model. We believe that mPEG × HER2-modified PLD can elevate susceptibility of ovarian cancer cells to PLD and eliminate residual cancer cells, thereby decreasing the frequency of relapse disease to achieve more effective targeted therapy of ovarian cancer. The design of the anti-tumor marker arm in the BsAb can also be changed to other antibodies to lead PLD or other mPEGylated drugs to target a various types of malignancies in the future.

Targeted liposomal drugs with enhanced endocytic ability can dramatically improve the drug susceptibility, cytotoxicity and therapeutic efficacy of malignancies. Wu and colleagues demonstrated that liposomal doxorubicin (LD) conjugated with pHCT74 peptide, which can specifically target to α-enolase (ENO1) on the surface of cancer cells and trigger endocytosis^28^, exhibits a half maximal inhibitory concentration (IC_50_) value of about twofold lower than nontargeted LD to colorectal cancer cells (HCT116) in vitro and significantly inhibits tumor growth by 80.1% as compared with nontargeted LD (65.8%) in HCT116-xenografted mice^28^. Lu et al. also suggested that anti-c-Met scFv (Ms20)-conjugated LD (Ms20-LD) can efficiently lead the liposomal drug to bind to c-Met receptor and induce an internalized mechanism and improve the IC_50_ to sixfold lower than unconjugated LD to lung cancer cells (H1993 and H441)^29^. The Ms20-LD was also proved to increase tumor accumulation rate 2.4 fold and therapeutic efficacy 1.9 fold in comparison with the LD group in human lung cancer cell (H460)-xenografted SCID mice^29^. Similarly, our mPEG × HER2-modified PLD exhibited higher endocytic ability (Fig. 2) and cytotoxicity (Fig. 3) in HER2-overexpressed ovarian cancer cells changing the IC_50_ from 870 to 160 ng mL^−1^ and significantly improving the therapeutic efficacy of SKOV-3-xenografted nude mice with minimal obvious toxicity (i.e. no significant body weight changed) as compared with non-targeted control BsAb-modified PLD (Fig. 5). We expect that the mPEG × HER2-modified PLD can significantly increase the safety of targeted PLD-treated individual and susceptibility of HER2^+^ ovarian cancer cells to PLD, reduce the number of residual cancer cells and further decrease the frequency of relapse disease after PLD treatment.

The anti-mPEG arm of the BsAb can be easily applied to different mPEGylated nanoparticle drugs to respond to the drug tolerance or resistance of ovarian cancer. PEG, especially mPEG, is a water-soluble, low immunogenic, non-toxic and biocompatible polymer that has been widely used in various therapeutic nanoparticles for cancer therapy^17,18,30^, such as mPEGylated liposomal-Doxorubicin (Lipo-Dox; Taiwan Liposome, Taiwan and Doxil/Caelyx; Johnson and Johnson, USA)^5,31^, mPEGylated liposomal-Irinotecan (Onivyde; Merrimack, USA)^32,33^ and PEGylated liposomal-Mitomycin C (PROMITIL) in Phase I clinical trials^34^. In our previous study, Kao et al. suggested that the mPEG × HER2 can confer HER2 specificity to various mPEG-NPs (Lipo/IR780, FeOdots or Qdot_565nm_) by a simple mixing procedure^25^ and enhance their targeting ability to SK-BR-3 (HER2^+^) tumor cells, but not MDA-MB-468 (HER2^−^) tumor cells^25^. We believe that the flexible strategy derived from one-step formulation of mPEG BsAb to mPEGylated nanoparticles can be easily transferred to other therapeutic options after the development of drug tolerance or resistance to ovarian cancer, performing uninterrupted attack on the residual cancer cells to prevent relapse disease.

The changeable design of the anti-tumor marker arm in our BsAb can properly shift to antibodies against different surface biomarkers to overcome the heterogenicity of ovarian cancer during treatment. Ovarian cancer is a heterogeneous tumor with five subtypes, high-grade serous (70%), low-grade serous (5%), endometrioid (10–15%), clear cell (10–15%) and mucinous (3%) tumor^2,9,35^. Several potential targets have been applied in clinical therapy (e.g., vascular epithelial growth factor (VEGF)^36–38^) or are under development in the preclincal stages (e.g., HER2^19^, programmed cell death protein-1 (PD-1)^39^ and mesothelin^40–42^, etc.). Our previous study demonstrated that the modular structure of the BsAb allows generation of BsAbs with specificity to different tumor antigens [i.e., epidermal growth factor receptor (EGFR)] and actively delivers mPEGylated nanoparticles (e.g., therapeutic agents or image probes) to SW480 (EGFR^+^) but not SW620 (EGFR^-^) tumor cells^25^. We anticipate that the changeable design of the anti-tumor marker arm of a BsAb can convert its antigen specificity to any potential surface biomarkers of different types of ovarian cancer for more comprehensive therapy.

In summary, non-covalent modification of mPEG × HER2 simply confers PLD with HER2 specificity and improves its tumor targeting, endocytic ability and drug susceptibility in ovarian cancer treatment. We suggest that the BsAb-modifying strategy has the following advantagies and potential: (1) easily provides tumor specificity to mPEGylated nanomedicines by one-step and non-covalently modification of the BsAb and avoids multi-directional modification of targeted agents caused by conventional chemical conjugation; (2) the elevated susceptibility of ovarian cancer to targeted PLD may efficiently eliminate residual cancer cells after debulking surgery and further prevent the frequency of relapse disease; (3) the changeable design of our BsAb can flexibly exchange either to different tumor targeted Abs or to different mPEGylated drugs against drug resistance of ovarian cancer. We expect that the wide applicability of BsAbs (mPEG × markers) can significantly improve the therapeutic efficacy, reduce the relapse frequency and thereby achieve good prognosis in ovarian cancer patients.

### Animal research

The study was carried out in compliance with the ARRIVE guidelines.

## Supplementary Information

Supplementary Information.
