# Supplementary material for: Enhancement of tumor tropism of mPEGylated nanoparticles by anti-mPEG bispecific antibody for ovarian cancer therapy
Source: Sci Rep. 2021 Apr 7;11:7598. doi: 10.1038/s41598-021-87271-2 (PMC8027450; doi:10.1038/s41598-021-87271-2)
Supplement: Supplementary file 1 — Supplementary Information. [file 41598_2021_87271_MOESM1_ESM.pdf]

Topic:

Enhancement of tumor tropism of mPEGylated nanoparticles by anti-mPEG bispecific antibody for ovarian cancer therapy

Wen-Wei Lin[1,2,3,4,13,***], Yi-An Cheng[3,5,***], Chia-Ching Li[3,5], Kai-Wen Ho[2], Huei-Jen Chen[2], I-Ju Chen[3,5], Bo-Cheng Huang[6], Hui-Ju Liu[2], Yun-Chi Lu[3,5], Chiu-Min Cheng[7], Ming-Yii Huang[8], Hung-Wen Lai[9,10,11,12,*], Tian-Lu Cheng[2,3,4,5,**]

[1]Department of Laboratory Medicine, School of Medicine, College of Medicine, Kaohsiung Medical University, Kaohsiung, Taiwan

[2]Graduate Institute of Medicine, College of Medicine, Kaohsiung Medical University, Kaohsiung, Taiwan

[3]Drug Development and Value Creation Research Center, Kaohsiung Medical University, Kaohsiung, Taiwan

[4]Department of Medical Research, Kaohsiung Medical University Hospital, Kaohsiung, Taiwan

[5]Department of Biomedical Science and Environmental Biology, Kaohsiung Medical University, Kaohsiung, Taiwan

[6]Institute of Biomedical Sciences, National Sun Yat-Sen University, Kaohsiung, Taiwan

[7]Department of Aquaculture, National Kaohsiung University of Science and Technology, Kaohsiung, Taiwan

[8]Department of Radiation Oncology, Cancer Center, Kaohsiung Medical University Hospital, Kaohsiung, Taiwan

[9]Endoscopy & Oncoplastic Breast Surgery Center, Changhua Christian Hospital, Changhua, Taiwan

[10]Division of General Surgery, Changhua Christian Hospital, Changhua, Taiwan

[11]Comprehensive Breast Cancer Center, Changhua Christian Hospital, Changhua, Taiwan

[12]Minimal invasive surgery research center, Changhua Christian Hospital, Changhua, Taiwan

[13]Department of Laboratory Medicine, Post Baccalaureat Medicine, College of Medicine, Kaohsiung Medical University, Kaohsiung, Taiwan

*Corresponding author. Director of the Endoscopic and Oncoplastic Breast Surgery Center, Comprehensive Breast Cancer Center, Changhua Christian Hospital, 135 Nanxiao Street, Changhua 500, Taiwan. Tel.: +886 4 7238595. Fax: +886 4 7233715. E-mail: 143809@cch.org.tw

**Corresponding author. Department of Biomedical and Environmental Biology, Kaohsiung Medical University, 100 Shih-Chuan 1st Road, Kaohsiung, 80708 Taiwan. Tel.: +886 7 3121101 2697. Fax: +886 7 3227508. E-mail: tlcheng@kmu.edu.tw

***These authors contributed equally to this work

Supplementary Information

Materials and Methods

Flow cytometry

Internalization of mPEG × HER2-modified nanoparticles into $HER2^+$ cancer cells was analyzed by adding 4 or 8 μg mL^{-1} mPEG × HER2-modified Lipo-DiD (HER2/Lipo-DiD) in staining buffer (PBS containing 0.05% (w/v) BSA) to 2×10^5 SKOV-3 cells for 40 min at 4°C. After extensive washing, the cells were transferred to fresh culture medium and incubated for 12 hours at 37°C. The surface BsAb-modified Lipo-DiD on the SKOV-3 cells was determined by sequentially adding 10 μg mL^{-1} 6.3 anti-PEG Ab for 30 min and 4 μg mL^{-1} FITC-conjugated goat anti-mouse IgG Fcγ. After washing, the FITC signal was measured with a Cytomics FC500 flow cytometer (Beckman Coulter, CA, U.S.A.).

In vitro cytotoxicity

3×10^3 SKOV-3 cancer cells were seeded in 48-well cell culture plate at 37°C for overnight. The next day, the cells were incubated with serum free medium (Control) or serial dilutions of Trastuzumab, mPEG × HER2 (100 μL $well^{-1}$) at 37°C. The cell viability was measured with the ATPlite™ luminescence assay system (PerkinElmer, Inc., Waltham, MA) after 96 hours post drug treatment. Results are expressed as percent inhibition of luminescence as compared with untreated cells and calculated by the following formula: % cell viability = 100 × (treated luminescence/untreated luminescence). The standard deviation for each points was averaged over four samples (n=3).

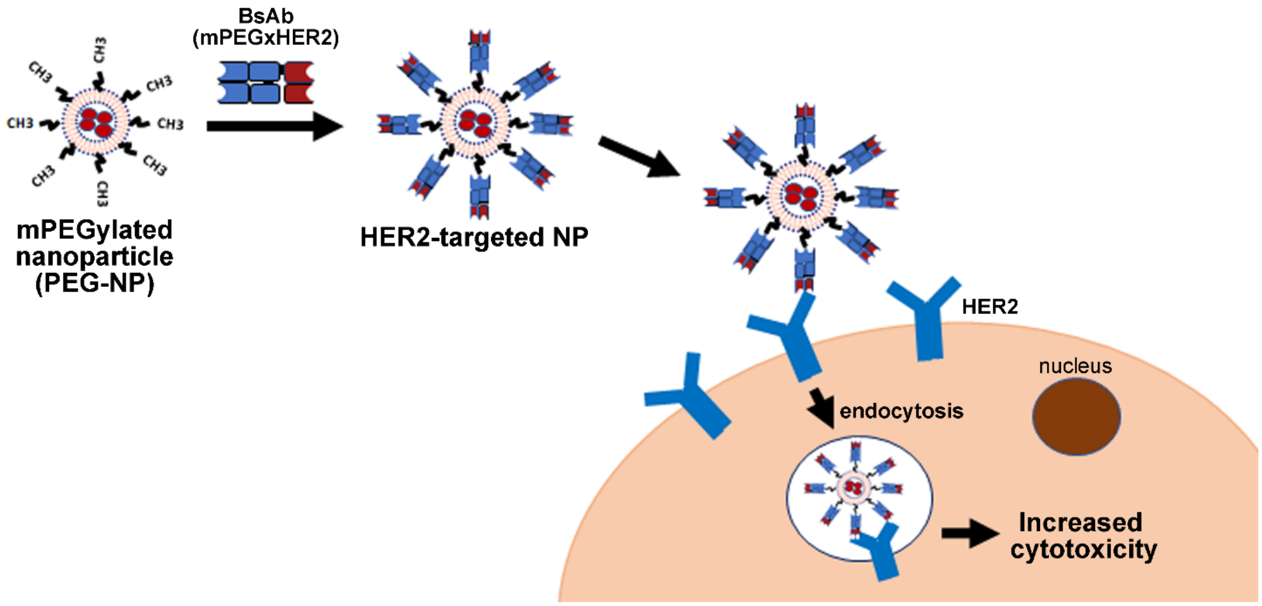

Supplementary Figure S1. mPEG × HER2 enhances targeting ability, endocytic efficiency and therapeutic effect of mPEGylated nanoparticle drugs in HER2^{+} ovarian cancer. mPEG × HER2 can confer HER2 specificity to mPEGylated nanoparticle drugs (e.g., PEGylated liposomal doxorubicin), specifically target HER2 antigen on the surface of cancer cells and efficiently trigger endocytosis for more effective ovarian cancer therapy. BsAb, bispecific antibody. mPEG, methoxyl polyethylene glycol.

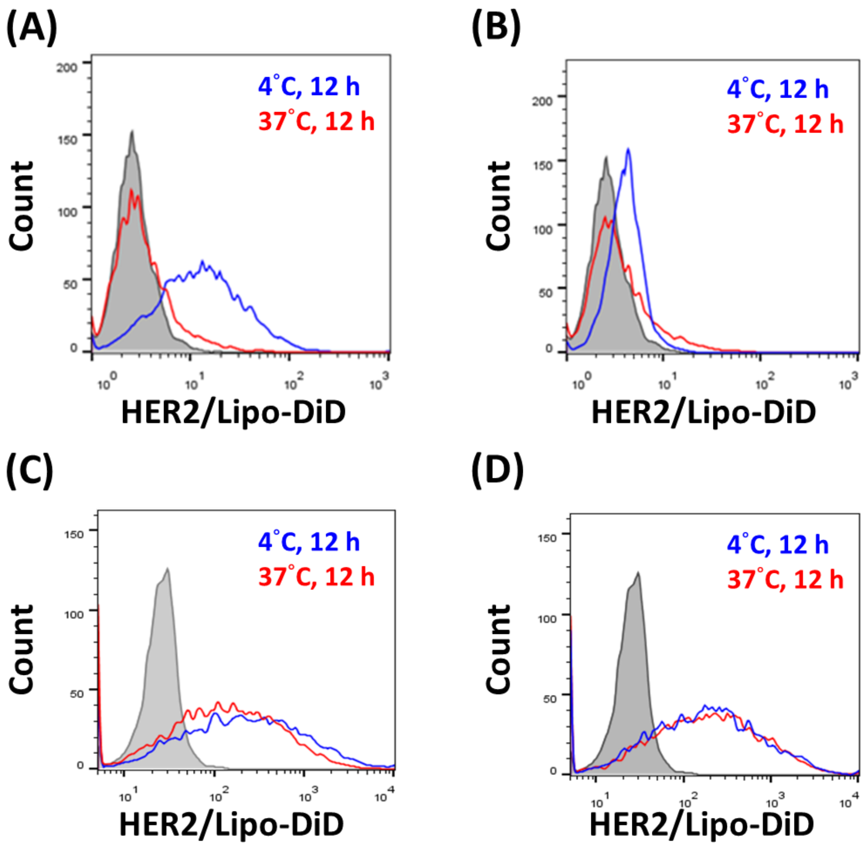

Supplementary Figure S2. The internalization of different dosage of mPEG × HER2-modified Lipo-DiD to SKOV-3 cells. The internalization ability of mPEG × HER2-modified Lipo-DiD was performed by incubating **(A and C)** 8 or **(B and D)** 4 μg mL^{-1} mPEG × HER2-modified Lipo-DiD (HER2/Lipo-DiD) to $HER2^{+}$ SKOV-3 cells at 4°C (blue) and then transferred cells to 37°C for 12 hours (red) and the surface bound mPEGylated nanoparticles were detected by staining with 6.3 anti-PEG antibody and FITC-conjugated secondary Ab and analyzed by Flow cytometry. The internalized Lipo-DiD in SKOV-3 cells were evaluated by monitoring the **(A and B)** decreasing level of surface FITC signal. The **(C and D)** total mPEGylated nanoparticles (including surface and internalized Lipo-DiD) were monitored by detecting the red fluorescent signal of Lipo-DiD. The gray peak on the graphs show mock staining with PBS containing 0.05% (wt/vol) BSA.

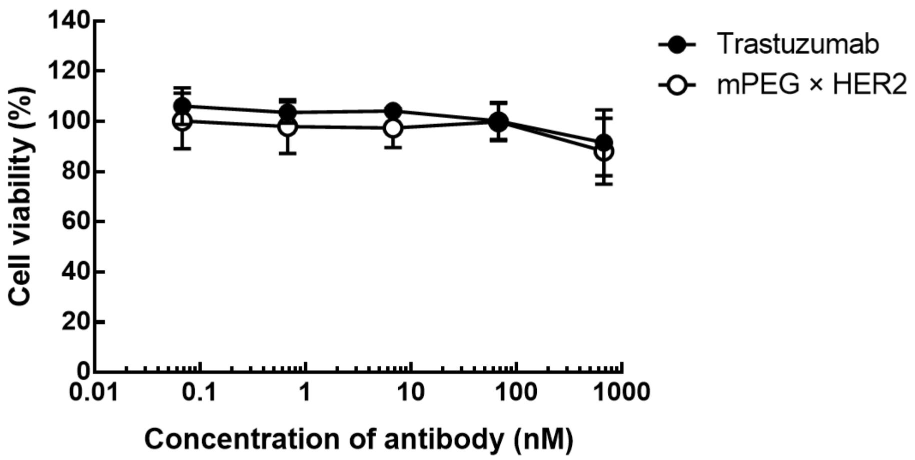

Supplementary Figure S3. The cytotoxicity of Trastuzumab and mPEG × HER2 BsAb to SKOV-3 cells. Anti-HER2 antibody (●, Trastuzumab) or mPEG × HER2 BsAb (○, mPEG × HER2) were incubated with SKOV-3 ovarian cancer cells for 96 hours. The cell viability was determined by ATPlite analysis and the mean luminescence values compared to untreated control cells (n = 3). Bars, SD.

Original blot version of Figure 3B

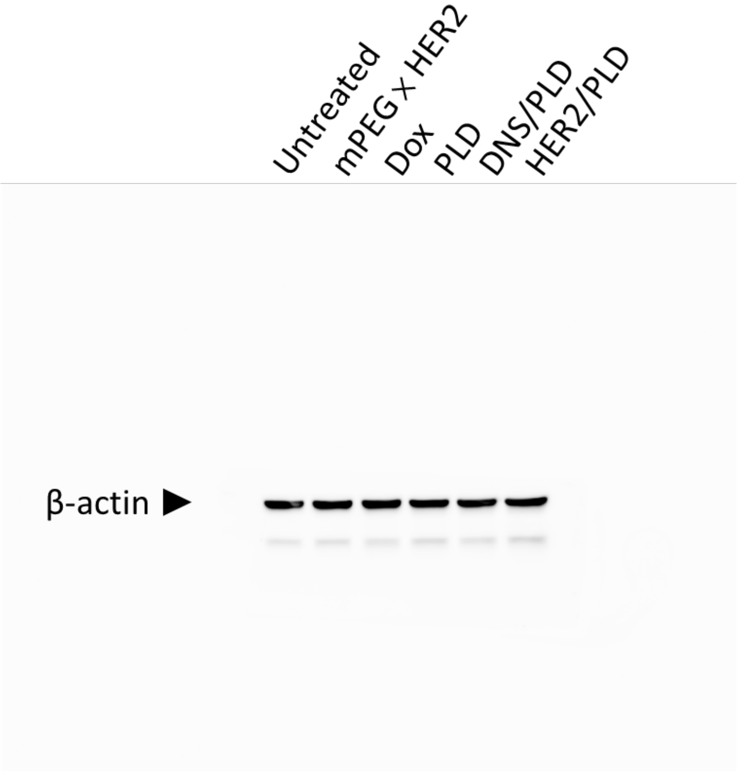

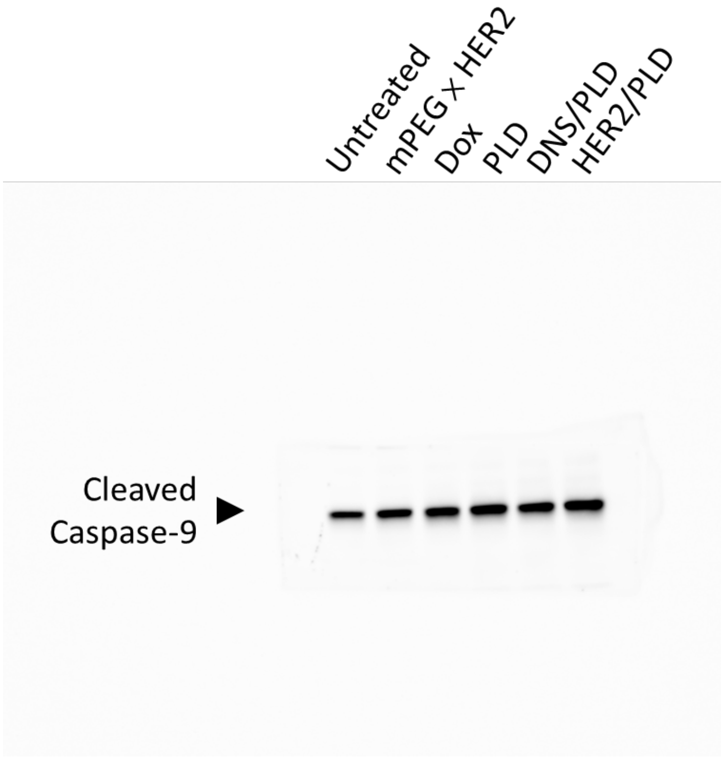

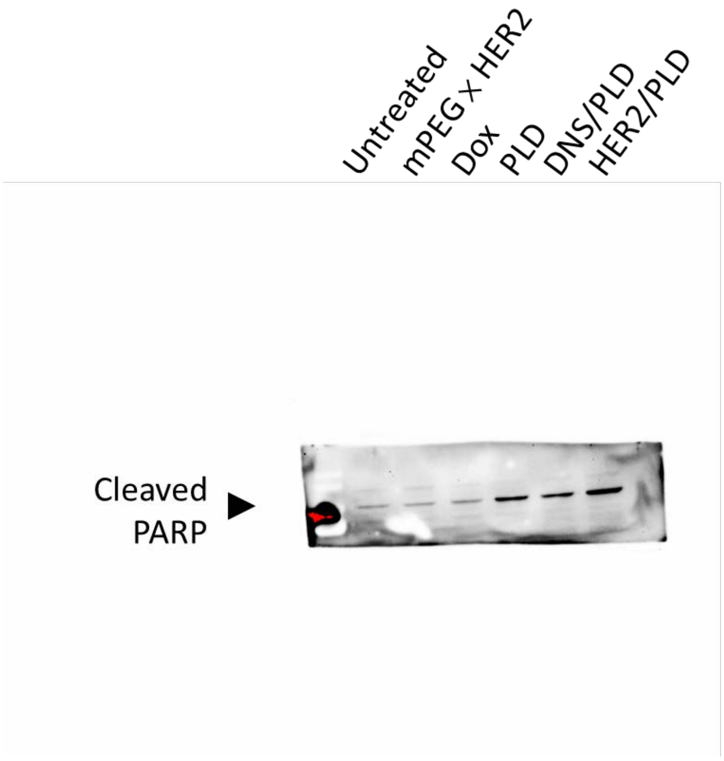
Untreated
mPEG × HER2
Dox
PLD
DNS/PLD
HER2/PLD
Cleaved
PARP
